# Effects of thymoquinone and memantine alone and in combination on memory and hippocampal morphology in rats with streptozotocin-induced Alzheimer’s disease

**DOI:** 10.55730/1300-0144.5653

**Published:** 2023-05-25

**Authors:** Şeyma ÖZSOY, Ziya ÇAKIR, Elif AKÇAY, Fikret GEVREK

**Affiliations:** 1 Department of Physiology, Faculty of Medicine, Tokat Gaziosmanpaşa University, Tokat, Turkiye; 2 Department of Oral and Dental Health, Faculty of Health Services Vocational School, Tokat Gaziosmanpaşa University, Tokat, Turkiye; 3 Department of Pathology, Faculty of Medicine, Tokat Gaziosmanpaşa University, Tokat, Turkiye; 4 Department of Histology, Faculty of Medicine, Tokat Gaziosmanpaşa University, Tokat, Turkiye

**Keywords:** Alzheimer’s disease, thymoquinone, memantine, streptozotocin

## Abstract

**Background/aim:**

Alzheimer’s disease (AD) is a progressive neurodegenerative disease. Thymoquinone (TQ) has broad biological functions, including antiinflammatory, antioxidant, neuroprotective properties. Memantine (MEM) is indicated for the symptomatic treatment of moderate to severe AD. We aimed to evaluate the effect of TQ alone or in combination with MEM on memory and hippocampal morphology in an STZ-induced rat AD model.

**Materials and methods:**

Thirty male rats were included in this study. The AD model was created by giving ICV STZ. The rats were divided into 5 groups (n = 6 each). Group 1 (control group): The rats received only ICV-STZ 3 mg/kg for 2 weeks. Group 2 (sham group): In addition to ICV STZ, 9% NaCl, 1 mL/day i.p. for 2 weeks of injection, was applied. Group 3 (TQ group): In addition to ICV STZ, rats received TQ 10 mg/kg i.p. for 2 weeks. Group 4 (MEM group): In addition to ICV STZ, rats were given MEM at a dose of 5 mg/kg for two weeks. Group 5 (TQ+MEM group): In addition to ICV STZ, this group was given TQ (10 mg/kg/day, i.p.) and MEM (5 mg/kg/day, i.p.) for 2 weeks. On the 15th day, passive avoidance learning (PAL) was applied to all groups. Then, rats were sacrificed, neurons in the hippocampal CA1, CA2, CA3 regions were evaluated.

**Results:**

Groups 3, 4, 5 had longer latency periods than groups 1 and 2. The neuron density in the CA1, CA2, CA3 regions had decreased in groups 1 and 2 compared to groups 3, 4, 5. There were significantly more neurons in groups 3, 4, 5 than in groups 1 and 2.

**Conclusion:**

We found that TQ alone and in combination with MEM showed ameliorative effects on memory and hippocampal morphology. TQ may offer a promising treatment strategy for AD.

## 1. Introduction

Alzheimer’s disease (AD) is a common neurodegenerative disease that is characterized by memory loss, cognitive dysfunction, and impairments in thinking skills and other behavioral abilities [1]. Pathologically, extracellular amyloid plaques and intracellular neurofibrillary tangles (NFTs) are seen [2]. Neurodegenerative changes in AD appear earliest in the hippocampus, and there is extensive NFTs accumulation in the early stages of the disease [3]. The N-methyl-d-aspartate receptor (NMDAR) plays a crucial role in synaptic transmission and synaptic plasticity, which is thought to form the basis of learning and memory and is central to neurotoxicity as well as the development and function of the nervous system. NMDAR activation has recently been implicated in AD-related synaptic dysfunction [4].

Memantine (MEM) is a low-affinity NMDAR channel blocker and has been used to treat moderate to severe AD [4]. Additionally, MEM protects from neuronal death accompanied by suppression of proliferation and activation of microglial cells in animal models of AD [5]. A metaanalysis of 12 randomized controlled trials evaluated the efficacy of MEM in the treatment of patients with AD, vascular dementia, and mixed dementia [6]. It was shown that MEM was superior to a placebo in improving cognitive function for mild to moderate AD and moderate to severe AD.

Thymoquinone (TQ) is the bioactive component of the essential oil of Nigella sativa (*N. sativa*) seeds. It is traditionally used in the Middle East and Southeast Asian countries because it has positive effects on health [7]. It has broad biological functions, including antiinflammatory, antioxidant, antihistamine, and antitumor properties [8]. In rats, a hydroalcoholic extract of *N. sativa* was shown to protect against synaptic plasticity, spatial learning, and memory disorders [9]. In addition, some animal and human studies have determined that *N. sativa* and its active ingredients can prevent neuron death and improve cognitive deficits such as memory loss [10, 11]. Streptozotocin (STZ), a glucosamine-nitrosourea compound formerly identified as an antibiotic, causes experimental diabetes due to its toxic effect on pancreatic beta cells. Human AD-like findings, such as decreased cognition and aggregation of cerebral Aβ fragments, tau protein, and Aβ deposits, occur when STZ is given by means such as intracerebroventricular (ICV) or intraperitoneal (i.p.) injection [12]. One of the accepted nontransgenic murine AD model in vivo is the STZ-induced model, which can display most features of AD in humans [13].

In this study, we aimed to evaluate the effect of TQ and MEM both alone and in combination on memory and hippocampal morphology in the STZ-induced AD model in rats.

## 2. Materials and methods

### 2.1. Animal ethics and housing conditions

Thirty adult male Wistar Albino rats (8–10 weeks old; 200–250 g) were included in the study. The animals were kept at 23 ± 1 °C on a 12-h light-dark cycle. Access to food and drinking water was given throughout the entire experiment. After receiving approval from the local ethics committee (2015-HADYEK-106), the current study was carried out in the Tokat Gaziosmanpaşa University Animal Laboratory.

### 2.2. Drugs and chemicals

Ketamine (10%) and xylazine (2%) were purchased from Alfasan Co (Holland). STZ, TQ, and MEM were obtained from Sigma Chemical Co. (St. Louis, MO, USA). All chemicals were dissolved in a 0.9% NaCl solution. The TQ and MEM were administered intraperitoneal (i.p.) and the STZ was administered intracerebroventricular (ICV) injection.

### 2.3. Experimental groups

The animals were divided into five groups, with six rats in each group, as follows:

Group 1 (control group): The rats received only ICV STZ 3 mg/kg for 2 weeks. No drugs were administered.
Group 2 (sham group): In addition to ICV STZ, 9% NaCl, 1 mL/day i.p. for 2 weeks of injection, was applied.
Group 3 (TQ group): In addition to ICV STZ, rats received TQ 10 mg/kg i.p. for 2 weeks, once a day.
Group 4 (MEM group): In addition to ICV STZ, rats were given MEM at a dose of 5 mg/kg i.p. for two weeks, once a day.
Group 5 (TQ+MEM group): In addition to ICV STZ, this group was given TQ (10 mg/kg/day, i.p.) plus MEM (5 mg/kg/day, i.p.) for 2 weeks, once a day.

Since the most effective dose of TQ used as neuroprotective in different experimental models in previous studies was 10 mg/kg, this dose was administered to rats [14, 15]. The MEM dose was also applied in the reference frame [16].

### 2.4. Creating the AD model

For the stereotaxic procedure, the animals were given general anesthesia by intraperitoneal (i.p.) administration of ketamine hydrochloride (75 mg/kg, Alfamine, Ege Vet, Alfasan International B.V. Holland) and xylazine hydrochloride (10 mg/kg, Alfazyne, Ege Vet, Alfasan International B.V. Holland). The scalps of the rats were placed in a Stoelting stereotaxic device (cutter bar set to −3.3 mm) (USA) and shaved and cleaned with povidone-iodine and ethanol. A midline incision was made, the skull was pierced, and 2.5 μL of STZ was administered to all rats in the right and left dorsal lateral ventricles according to the Paxinos and Watson atlas (AP = −0.8mm, L = ±1.6 mm, DV = −4.2 mm) [17]. The rats in group 2 were injected with a 0.9% NaCl solution. All injections were administered with a 5-μl Hamilton microinjector for 60–90 s and then in situ for 5 min to allow the drug to be distributed. After the drug had diffused, the syringe was slowly withdrawn. Room temperature was controlled during the procedure, and body temperature was maintained at 37 °C. After the procedure, the animals were monitored daily for behavior and health.

### 2.5. Passive avoidance learning (PAL) test

The passive avoidance learning (PAL) test is based on the fact that subjects learn to avoid an environment in which they have previously received a negative stimulus. The PAL test is used to assess learning and short- and long-term memory in rats and mice. Additionally, both hippocampus-dependent contextual memory and amygdala dependent fear conditioning emotional memory can be evaluated by this method [18]. Two weeks after the treatment period, PAL tests were carried out in the groups. For this, a PAL box with a size of 20 × 20 × 20 cm with dark and light sections was used. The rats were placed in the bright chamber of a two-compartment box. After a 10-s acclimatization time, the door between the light and dark chambers was opened. After the animal passed into the dark area, the door was closed, and an electric shock was applied to the animal for 3 s. The time it took for the animal to enter the dark zone was considered the latency period. Animals that did not enter the dark zone for more than 5 min were excluded from the study. After 24 h, the animals were placed back into the illuminated area with the door open. The time for the animal to enter the dark zone was evaluated and was considered the latency time. Latency cutting time was determined as 5 min [19].

### 2.6. Histological evaluation

**Figure 1:**
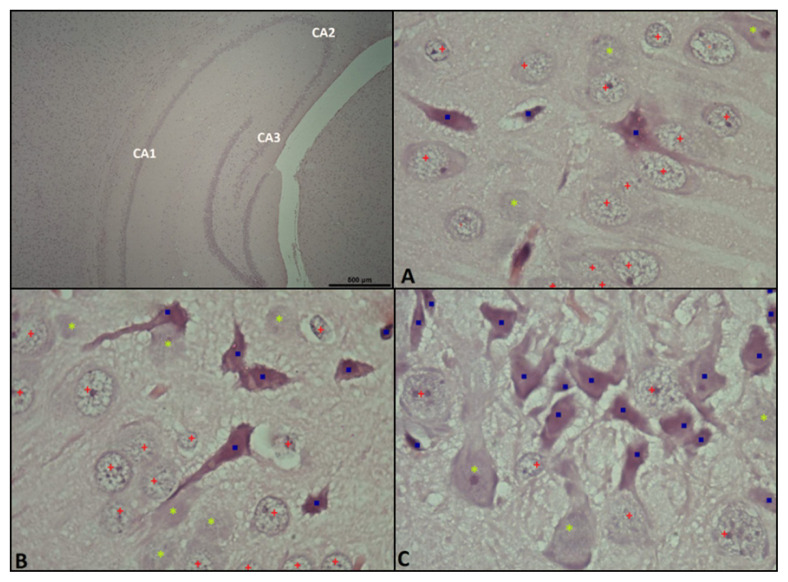
The histopathological changes in the cornu ammonis (CA) sections of the hippocampal tissue were evaluated in the tissue sections. CA1, CA2, and CA3 hippocampal regions were stained with H&E stain (×40 and ×100 magnification). A, B, and C are sample histologic images from a representative count of CA1, CA2, and CA3 regions, respectively. The blue square dot marks strongly dark stained cells, the light green star marks slightly dark stained cells, and the red square marks normally light stained cells.

After the PAL procedure, all rats were sacrificed by cervical decapitation and their brain tissues were removed for histopathological and biochemical evaluation. Brain tissue samples were taken from all animals and placed in 10% formalin. After the brain hemispheres were removed from the skull, one of the hemispheres (right) was fixed in a 4% buffered neutral formaldehyde solution for three days. After fixation, the brains were embedded in paraffin blocks in the same orientation and blocked. The parts of the blocked tissues where the hippocampus was fully visible (by taking 4–5 μm thick sections) were placed on slides. Tissue sections were placed on an adhesive slide and stained with the hematoxylin-eosin protocol. The prepared preparations were covered with a coverslip and made ready for neuron counting. These preparations were analyzed with a computer-assisted research light microscope (Nikon Eclipse, E 600, Tokyo, Japan) and the Nis element program (Hasp ID: 6648AA61; Nikon). The histologist was blind to groups when scoring the samples. Histopathological changes in the cornu ammonis (CA) sections of the hippocampus tissue were evaluated in tissue sections (Figure 1). Analyses were performed by counting the cells in each area separately, taxonomically, according to the staining intensities of nerve cells from each region in the CA1 (Figure 1A), CA2 (Figure 1B), and CA3 (Figure 1C) regions of the hippocampus on consecutive average sections from each individual. The counts in each area were performed by analyzing 100× objective images. The criteria used in grading the staining of neurons are given in Table [20].

Represented by 0 here are normal and undamaged neurons with normal staining and prominent euchromatic nuclei. The neurons represented by 1 represent moderately damaged neurons, and those represented by 2 represent severely damaged neurons stained very dark. The weighted average values of the staining intensities for each individual of the neuron counts, which were counted according to the staining intensities in 100× images with the help of the NIS Elements program, were calculated.

### 2.7. Statistical analysis

Statistical analyses were performed with the SPSS 22.00 program. In order to compare the differences between the groups, they were compared with the one-way ANOVA test and then with the Tukey test from the posthoc LSD tests. The results were presented as the mean (mean) and standard error (SEM). p values of <0.05 were considered statistically significant.

## 3. Results

### 3.1. PAL results

**Figure 2:**
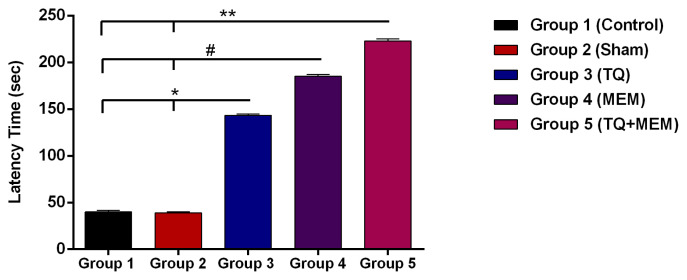
The mean latency time for all groups. * p < 0.05, group 3 vs. groups 1 and 2; # p < 0.01, group 4 vs. groups 1 and 2; ** p < 0.001, group 5 vs. groups 1 and 2.

PAL latency time was evaluated in all groups. There was no statistical difference between the groups 1 and 2 (40 ± 1.5 s and 39 ± 0.96 s; p > 0.05, respectively). Groups 3 and 4 had longer latency periods than the groups 1 and 2 (143.33 ± 1.58 s and 185 ± 1.88 s; p < 0.05 and p < 0.01, respectively). Group 5 had a longer latency time than groups 1 and 2 (group V: 222.83 ± 2.21 s) (p < 0.001). Figure 2 shows mean latency time as an index of memory retention after STZ induction into the lateral ventricles.

### 3.2. Histological results

We evaluated the effects of TQ and MEM on neurons in hippocampal CA1, CA2, and CA3 regions. Dark-stained neurons were evaluated as severely damaged, and neurons that showed normal light were considered normal. Neuronal loss in the CA1, CA2, and CA3 regions was greater in the control and sham groups compared to the other groups. Groups 3, 4, and 5 had higher neuron counts than the control group (p < 0.001). There was no statistical difference between groups 3, 4, and 5 in terms of neuronal activity (p > 0.05).

**Figure 3:**
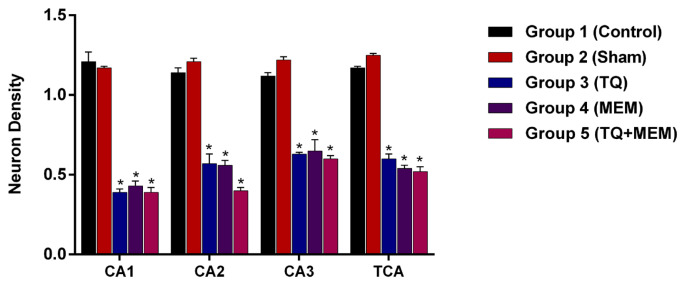
The staining densities scores of neurons in hippocampal CA1, CA2, and CA3 regions of groups. * p < 0.001, groups 3, 4, and 5 compared to groups 1 and 2.

When we evaluated the hippocampus CA regions, it was found that the neuron staining intensities did not change in groups 1 and 2. Group 1 was statistically different from the treatment groups (p < 0.001). Neuronal damage was less in groups 3, 4, and 5 compared to group 1 (p < 0.001). Groups 3, 4, and 5 were statistically similar to each other (p > 0.05). Results are shown in Figure 3.

## 4. Discussion

AD, a neurodegenerative disease, is associated with neurobehavioral deterioration, dementia, and neuronal cell loss in the brain [21]. It affects 5%–8% of the population over the age of 60 [22]. There are approximately 50 million patients worldwide. Aside from the gradual deterioration of daily activities, AD causes a cognitive decline that impairs memory, orientation, and reasoning. Physiopathologically, the disease includes neurofibrillary degeneration, neuroinflammation, reactive gliosis, oxidative stress (OS), and neuronal loss. Current pharmacotherapeutics for AD are based on relieving symptoms, preserving mental abilities, and delaying the progression of neurodegeneration.

MEM, which is used in the treatment of AD, blocks NMDAR, reduces hyperexcitability, and protects neurons and their functions [23]. MEM has appropriate safety and tolerability limits. There are in vitro [24, 25] and in vivo studies [26, 27], showing that MEM protects neurons from the toxic effects of glutamate by regulating glutamatergic system homeostasis [23]. Some preclinical studies have reported that MEM leads to a decrease in Aβ brain levels or reduces amyloid plaque burden in preclinical mice models of AD, which has a positive effect on memory development [28]. In addition, MEM was effective in improving short-term memory deficits in APP/PS1 mice, according to the results of the new object recognition test [29]. Stazi et al. reported that a 4-month chronic oral MEM treatment improved memory performance and reduced hippocampal CA1 neuron loss [30]. Matsunaga et al. reported that AD patients tolerated MEM monotherapy well and that it improved cognition, behavior, activities of daily living, general functionality, and dementia [31]. However, it was also noted that the size of the effect was small. Chronic administration of MEM has been shown to protect against hippocampal neuron damage induced by intracerebroventricular injection of quinolinic acid in rats [32]. In the β-amyloid-induced neurodegeneration model study, neurodegeneration was shown to be reduced in MEM-treated rats compared to vehicle-treated rats [33].

Plants are used in traditional medicine as a major source of active compounds. *N. sativa* is considered a medicinal herb with some religious uses. It has been called “the cure for all diseases except death” and “The Blessed Seed” in history [34]. TQ, the main ingredient of *N. sativa*, is effective in the treatment of various diseases, such as neurodegenerative disorders, coronary arterial, respiratory, and urinary system diseases [35]. OS plays an important role in the pathogenesis of AD. An increase in free radical production and a decrease in the activity of antioxidant enzymes occur in the central nervous system and peripheral tissues of patients with AD [36]. TQ has high antioxidant activity and antiinflammatory properties. Imam et al. studied the effects of black seed oil on cognitive function and corticohippocampal neural changes in male Wistar rats. In this study, cognitive impairment was induced in rats with scopolamine. Black seed oil improved memory impairment by reducing latency and corrected cognitive impairment induced by scopolamine [37]. An in vitro study showed that, after exposure to a neurotoxic concentration of Aβ, TQ can increase the number of surviving cells in cultured hippocampal neurons [38]. A study demonstrated that TQ has neuroprotection potential against Aβ1-42 in rat hippocampal and cortical neurons. Impairment of learning and memory in AD is an important cause of dementia [39]. According to this study, the functional improvement provided by TQ was related to an increase in neurogenesis and an improvement in IFN-*γ* levels in the AD rat model. It was reported that TQ protected against propylthiouracil-induced memory impairments in rats [40]. Kanter showed that TQ and *N. sativa* resulted in morphological improvement in neurodegeneration in the hippocampus after chronic toluene exposure in rats [41]. Hajipour et al. showed that TQ treatment can ameliorate the negative effects of thioacetamide-induced hepatic encephalopathy on learning and memory, increase spontaneous neuronal activity, inhibit apoptosis, and reduce neuroinflammation [42].

In this study, we examined the effects of TQ and MEM alone and in combination in an AD model. To the best of our knowledge, this is the first study to evaluate the combination of TQ and MEM. PAL test results showed that treatment of TQ, MEM or both had a positive effect on learning and memory. According to our results, TQ improved the learning function, and accordingly the latency time was prolonged. The hippocampus, an important component of the limbic lobe, contains the dentate gyrus (DG) and CA regions (CA1, CA2, CA3, and CA4) [43]. The CA regions contain densely packed pyramidal cells. In AD, neurofibrillary tangles first accumulate in the CA1 region of the hippocampus, then gradually affect the subiculum, CA2, CA3, and DG [44]. One of the most affected areas of the brain in patients with advanced AD is the hippocampus. The consistency of hippocampal histopathology has led to AD being described as ‘hippocampal dementia’ [45]. One study investigated the relationship between neuronal loss and hippocampal volume in 11 autopsy-confirmed AD patients and 11 nondementia controls [46]. The results of this study showed strong correlations between the number of neurons, hippocampal volume, and brain volume. In AD, neuron number and volume measurements show a marked decrease. In this study, we examined histologically the brain tissue in groups. According to our results, significantly more neuronal loss was observed in CA1, CA2, and CA3 regions in groups 1 and 2. However, there was a significant increase in the number of neurons in hippocampal CA1, CA2, and CA3 regions in groups 3, 4, and 5. The results of our study emphasize that TQ has the effect of improving memory and hippocampal morphology, which may be important in the recovery of neurological diseases.

## 5. Conclusion

We examined the effects of TQ and MEM alone or together on memory and hippocampal morphology. The STZ was used to stimulate the experimental model of the AD model. Although drugs are used to improve the cognitive and behavioral symptoms of AD, alternative treatment approaches need to be developed. These findings shed important light on the relationships between neurodegenerative disease and TQ. There are several limitations, however, that must be taken into account. It was not determined whether TQ treatment had an antiapoptotic, antiinflammatory, or antioxidant effect in the hippocampal area. In addition, sex-related differences could not be distinguished in the AD rat model. As a result, the neuroprotective effect of TQ has been demonstrated in this study, increasing the scientific value of TQ for the prevention of neurodegenerative diseases. The easy accessibility and ease of application of herbal medicines will enable them to be used in the treatment of many neurodegenerative diseases. Further studies are needed in which analyses are carried out to demonstrate the change at the molecular level.

## Figures and Tables

**Table:** Criteria used in the evaluation of neuron damage.

*Score*	*Neuron staining criteria*
*0*	Normal staining
*1*	Light intense staining
*2*	Severe intense (dark) staining

Normal and undamaged neurons with normal staining and prominent euchromatic nuclei are represented by 0. The neurons indicated by 1 are moderately injured neurons, these shown by 2 are very darkly stained and highly damaged neurons.
